# Functional Validation of Hydrophobic Adaptation to Physiological Temperature in the Small Heat Shock Protein αA-crystallin

**DOI:** 10.1371/journal.pone.0034438

**Published:** 2012-03-29

**Authors:** Mason Posner, Andor J. Kiss, Jackie Skiba, Amy Drossman, Monika B. Dolinska, J. Fielding Hejtmancik, Yuri V. Sergeev

**Affiliations:** 1 Department of Biology, Ashland University, Ashland, Ohio, United States of America; 2 Department of Zoology, Miami University, Oxford, Ohio, United States of America; 3 Ophthalmic Genetics and Visual Function Branch, National Eye Institute, National Institutes of Health, Bethesda, Maryland, United States of America; University Medical Center Groningen, University of Groningen, The Netherlands

## Abstract

Small heat shock proteins (sHsps) maintain cellular homeostasis by preventing stress and disease-induced protein aggregation. While it is known that hydrophobicity impacts the ability of sHsps to bind aggregation-prone denaturing proteins, the complex quaternary structure of globular sHsps has made understanding the significance of specific changes in hydrophobicity difficult. Here we used recombinant protein of the lenticular sHsp α A-crystallin from six teleost fishes environmentally adapted to temperatures ranging from -2°C to 40°C to identify correlations between physiological temperature, protein stability and chaperone-like activity. Using sequence and structural modeling analysis we identified specific amino acid differences between the warm adapted zebrafish and cold adapted Antarctic toothfish that could contribute to these correlations and validated the functional consequences of three specific hydrophobicity-altering amino acid substitutions in αA-crystallin. Site directed mutagenesis of three residues in the zebrafish (V62T, C143S, T147V) confirmed that each impacts either protein stability or chaperone-like activity or both, with the V62T substitution having the greatest impact. Our results indicate a role for changing hydrophobicity in the thermal adaptation of α A-crystallin and suggest ways to produce sHsp variants with altered chaperone-like activity. These data also demonstrate that a comparative approach can provide new information about sHsp function and evolution.

## Introduction

Small heat shock proteins (sHsps) play a central role in protein homeostasis by preventing the stress and age-related aggregation of denaturing proteins through their chaperone-like activity [1], [2], [3]. Small Hsps also have non-chaperone roles in routine cellular functions such as the regulation of apoptosis, cytoskeletal rearrangement and the maintenance of cell membrane fluidity [4], [5]. An increasing number of studies have shown that sHsp dysfunction is related to diseases of the nervous, muscular and visual systems, and that changes in sHsp expression are tied to multiple types of cancer [6], [7], [8], [9]. Many studies into sHsp function have focused on the α-crystallins, a group of structural proteins in the vertebrate ocular lens that are also expressed in multiple non-lenticular tissues [10], [11] and play the same protective and housekeeping roles as other sHsps [2], [12]. Mammals express two α-crystallins (αA- and αB-crystallin) that form large oligomers containing 10–40 subunits [13].

Biophysical approaches have been used to investigate how α-crystallins and other sHsps interact with compromised target proteins to prevent aggregation [14], [15], [16], [17] and attempts have been made to detail the functional importance of specific residues and regions [18], [19], [20]. Alphacrystallin oligomers are thought to prevent protein aggregation by releasing subunits that block the attraction of hydrophobic regions on partially unfolded proteins [21], although there is some evidence that chaperone-like activity results from binding of the intact oligomer [22]. Either way, the dynamics of oligomerization seem to play a prominent role in the regulation of sHsp function [17], [23]. Hydrophobicity of the sHsp affects protective chaperone activity by both influencing the stability of the oligomer, thus affecting “subunit exchange", as well as changing how detached subunits interact with their compromised target proteins [24], [25]. The effect of hydrophobicity changes are highly context dependent, such that it is difficult to predict how an alteration of hydrophobicity at specific residues might affect chaperone activity. In addition to hydrophobicity, temperature can also alter oligomer stability and subunit/target protein binding [26], [27]. A previous study showed that the stability of diverse vertebrate lens crystallins adapts to different environmental temperatures [28], yet no study to our knowledge has used a similar comparative approach to identify individual amino acid changes that may adjust sHsp stability or chaperone-like activity. We hypothesized that vertebrate α-crystallins adapted to a range of environmental temperatures would exhibit changes in hydrophobicity that alter both oligomer stability and protein binding activity, providing a unique model for investigating hydrophobic influence on α-crystallin/sHsp function.

To identify specific changes in hydrophobicity affecting sHsp function we compared αA-crystallins from ectothermic teleost fishes adapted to environmental temperatures spanning −2°C to 40°C. While the zebrafish (*Danio rerio*) contains two αB-crystallin proteins with divergent structures and function [29], [30], the single zebrafish αA-crystallin has not changed significantly from its mammalian ortholog [31], [32], [33]. This conservation makes teleost fishes a valuable model group for comparative studies of αA-crystallin structure and function. We previously showed that zebrafish and Antarctic toothfish α-crystallins have reduced thermal stability and increased chaperone-like activity compared to their mammalian orthologs over a broad range of temperatures, suggesting adaptation to their lower environmental temperatures [31], [34]. In this present study we used six bony fish species differing in mean physiological temperature as a model group to identify specific residues that have evolved to alter αA-crystallin function. We found that αA-crystallin stability and chaperone-like activity correlated with species' environmental temperature. Comparison of the six αA-crystallin amino acid sequences identified three specific amino acid residues varying in hydrophobicity that could contribute to this correlation. Site directed mutagenesis of zebrafish αA-crystallin confirmed a significant functional role for two of these three residues. These findings provide insights into how hydrophobicity affects sHsp function and suggests mechanisms by which αA-crystallin has adapted to different environmental temperatures. We also show for the first time that comparisons of naturally evolved sHsps can contribute to the design of functional sHsps with altered chaperone-like activity.

## Results

### αA-crystallin chaperone-like activity and thermal stability correlate with each species' physiological temperature

**Figure 1:**
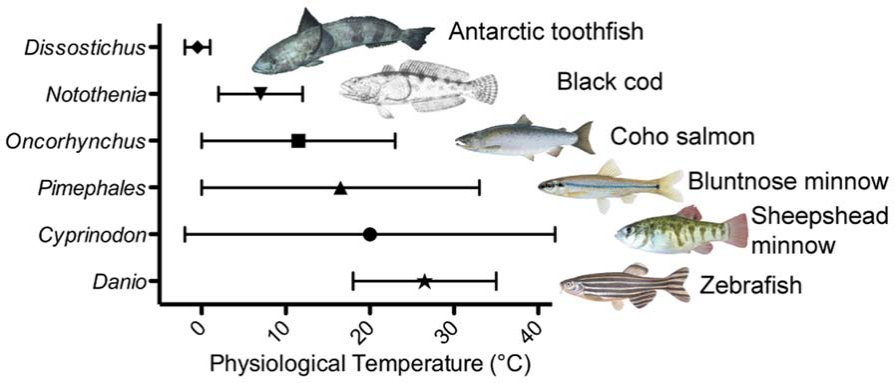
Physiological temperatures of species used in this study. Physiological range and average temperature are indicated for each species. Genus is noted on the ordinate with common names provided to the right of each picture.

**Figure 2:**
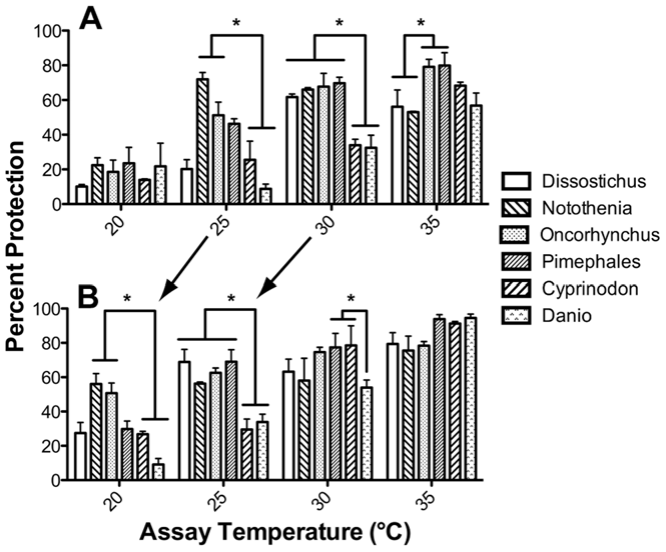
Chaperone-like activity of αA-crystallin reflects species' physiological temperature. Higher percent protection indicates greater ability to prevent chemically induced aggregation of insulin (A) or lactalbumin (B) at four different temperatures. Species are arranged left to right from coolest to warmest mean physiological temperature. When compared at the same assay temperature, αA-crystallins from cooler bodied species generally showed greater chaperone-like activity, except at 35°C with insulin where cooler adapted proteins may be thermally stressed. Arrows highlight similar patterns produced by both target proteins, with a 5°C shift in temperature. Error bars indicate standard error of the mean (n = 3) and asterisks indicate statistically significant differences (ANOVA, Bonferroni posttest, p-value<0.05).

Orthologous αA-crystallin genes from six bony fish species (Fig. 1) differing in physiological temperature were cloned and recombinant protein produced. Assays of each αA-crystallin's chaperone-like activity showed that the ability to prevent the aggregation of denaturing proteins was correlated with the physiological temperature of each species (Fig. 2). For example, percent protection against insulin aggregation at 25°C and 30°C was statistically significantly higher in species from lower environmental temperatures (Fig. 2A). The exception to this pattern was the coldest species, the Antarctic toothfish *Dissostichus mawsoni*, which exhibited relatively low protection at 25°C. The reduction in chaperone-like activity with insulin at 35°C in the two coolest bodied fishes (*Dissostichus* and *Notothenia*) relative to the rest could be due to excessive thermal denaturation. An almost identical pattern was seen when lactalbumin was used as a target protein but shifted 5°C lower, suggesting that the correlation between chaperone-like activity and the physiological temperature of each species is due to the αA-crystallins themselves and not a byproduct of each target protein (Fig. 2B). Differences between cold and warm-adapted species are less obvious as assay temperatures increased due to the various αA-crystallins approaching a ceiling of 100% protection with the ratio of crystallin to target protein used in these specific assays.

**Figure 3:**
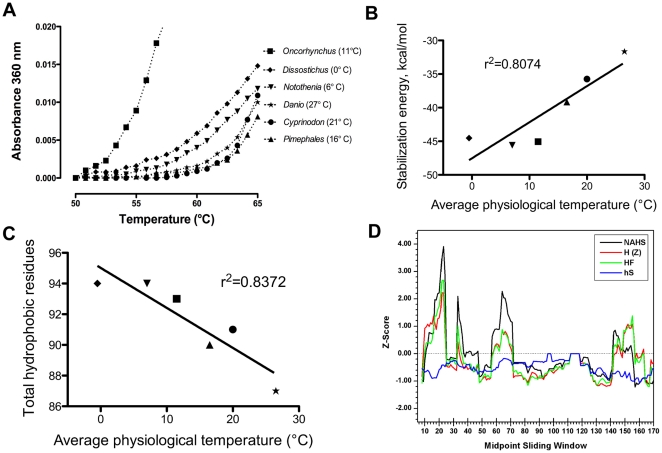
Thermal stability and hydrophobicity of six fish αA-crystallins was correlated with physiological temperature. Both direct measurement and computer-based estimation of protein stability indicated that αA-crystallins from wamer bodied species are more thermally stable. (A) Each crystallin was incubated at 50°C at time 0 and then heated 2.5°C every 15 minutes. Initiation of denaturation and protein aggregation occurred at lower temperatures with αA-crystallins from the three most cold-adapted species. This method for measuring thermal stability does not assess absolute stability of each crystallin, but does reflect relative stability. (B) In silico calculation of each protein's stabilization energy using homology modeling based on a bovine αA-crystallin monomeric structure (PDB ID: 3L1E) showed a strong correlation between global protein stability and physiological temperature. (C) The total number of hydrophobic residues in the six fish αA-crystallin domains decreases with increasing physiological temperature. The total number of hydrophobic residues was determined using the SAPS (http://www.ebi.ac.uk/Tools/saps/index.html). (D) Selection on physiochemical properties in a phylogenetic context with TreeSAAP detected regions of αA-crystallin in which several hydrophobic parameters (NAHS = Normalized Average Hydrophobicity Scales, H (Z) = Hydrophobicity, HF = Hydrophobicity Factor, hS = hydrophobicity Scales) are under positive natural selection. Sliding window of 15 with a step of one was used; midpoint mean is displayed.

The ability of α-crystallins to bind denaturing proteins and protect them from aggregation requires structural flexibility as chaperone-like activity involves both the exchange of subunits from the α-crystallin oligomer and the exposure of hydrophobic residues [21], [35]. We examined whether differences in chaperone-like activity between fish αA-crystallins adapted to disparate thermal environments were correlated with variations in thermal stability. We found that αA-crystallins from the three species with the lowest mean environmental temperatures (*Dissostichus*, *Notothenia* and *Oncorhynchus*) had lower thermal stability than those from the three warmest species (Fig. 3A). However, the correlation between thermal stability and environmental temperature is not exact, as the temperate *Oncorhynchus* αA-crystallin showed the lowest stability, while the temperate *Pimephales* ortholog was the most stable. Overall, these data suggest that while αA-crystallin stability has adapted to divergent environmental temperatures, it is not the sole determinant of chaperone-like activity. This would explain how *Pimephales* αA-crystallin can exhibit higher chaperone-like activity than that of the con-familial *Danio* ortholog even with similar global stabilities. Furthermore, the thermal stability assay used only measures global protein stability, and not the flexibility of individual subunits needed to expose hydrophobic region for target protein binding.

To understand the role of sequence variability in maintaining protein stability we modeled the structure of each fish αA-crystallin's conserved “alpha crystallin" domain (residues 59–153 of the *Danio* protein) using the bovine αA-crystallin monomer as a structural template. Modeled structures were refined, equilibrated using molecular dynamics, and protein stabilization energies were estimated as described in Methods. We found a significant correlation (R^2^ = 0.8074) between each species' mean environmental temperature and the stabilization energy of its αA-crystallin, with greater relative stability in proteins from warmer species (Fig. 3B).

### αA-crystallin hydrophobicity is related to physiological temperature

Previous studies have shown changes in hydrophobicity can alter both the stability and chaperone-like activity of α-crystallins and that these effects are location dependent (see [24] for review). We found a strong inverse correlation between the total number of hydrophobic residues and the average physiological temperature of each of the six species, with warmer bodied species containing a smaller number of hydrophobic residues (Fig. 3C). This result suggests a possible role for hydrophobic residues in the evolutionary adaptation of α-crystallin to different thermal environments. Using a phylogenetic approach we identified specific αA-crystallin regions in which hydrophobic properties are likely to be undergoing positive natural selection. The selective evolution of these properties would suggest that these specific regions are involved in adaptation to different environmental conditions, such as temperature. The computer program TreeSAAP was used to compare the αA-crystallin sequences from different taxa to create an ancestral sequence from which to evaluate any natural selection in extant species. We then observed positive selection in four hydrophobic properties along an alignment of fish αA-crystallins, with significant deflections in the following four regions (p≤0.05; numbers indicate residues in the zebrafish sequence): 10∼25; 32∼37; 56∼72; 141∼157 (Fig. 3D).

### Homology modeling identifies three amino acid substitutions with potential effects on αA-crystallin function

**Figure 4:**
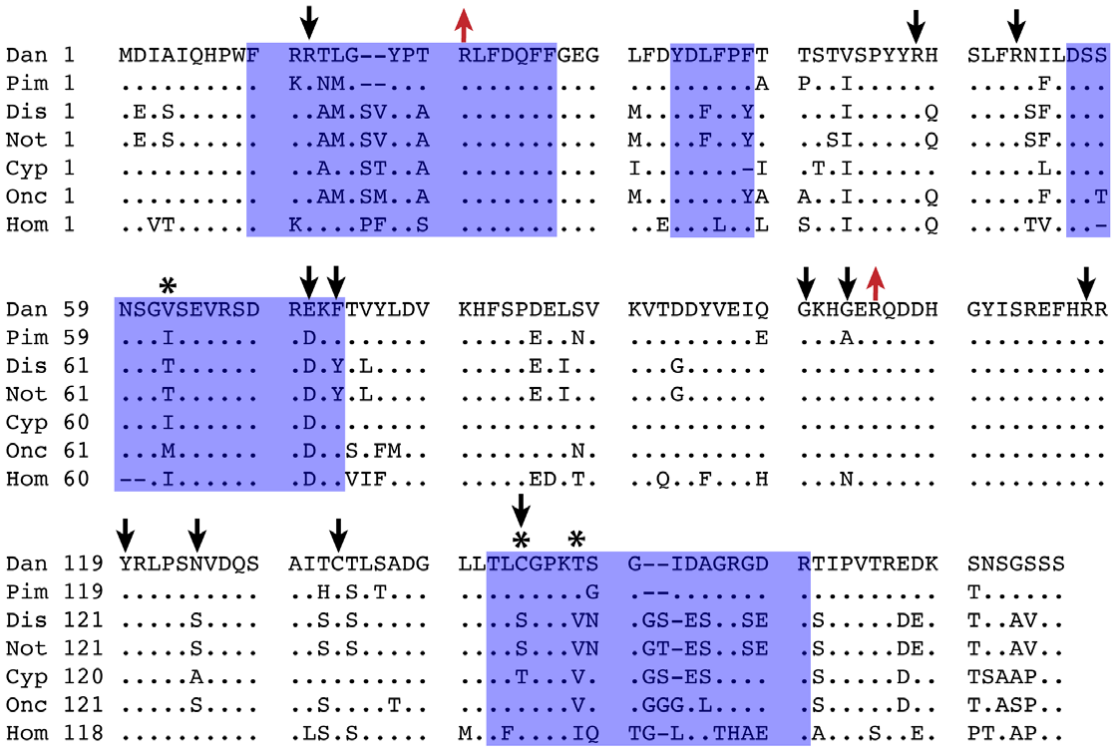
Multiple sequence alignment of αA-crystallin amino acid sequences from six bony fish species and human. Dots indicate residues identical to the zebrafish sequence. Arrows indicate residues that have undergone site-directed mutational analysis in the literature (see Table S1). Downward facing arrows are sites that have been modified to decrease chaperone activity while upward facing orange arrows are sites that have been modified to increase chaperone-like activity. Purple shaded boxes are the center (midpoint of the window (Fig. 3D)) of the regions in which hydrophobicity has undergone positive natural selection as identified by TreeSAAP analysis (Fig. 4D). Asterisks indicate three variants (V62T, C143S, T147V) examined in this study. These variants alter hydrophobicity at each residue and were predicted to influence chaperone-like activity based on homology modeling using bacterial sHsp crystal structures. Species shown are (with accession numbers): *Cyprinodon variegatus* (Cyp; HQ111072), *Danio rerio* (Dan; NP_694482), *Dissostichus mawsoni* (Dis; ABA61342), *Homo sapiens* (Hom; CAG28619), *Notothenia angustata* (Not; HQ111073), *Oncorhynchus kisutch* (Onc; HQ111071), *Pimephales notatus* (Pim; HQ111070).

We produced an alignment of the six fish αA-crystallin amino acid sequences and their human ortholog to identify variations that could contribute to differences in chaperone-like activity and thermal stability (Fig. 4). Identity between the fish sequences ranged from 76.8% to 98.9% (*Dissostichus* and *Notothenia* most similar), and sequence identity between the fishes and human sequence ranged from 68.4% to 73.3%. Variation between the six fish species occurred at 56 of 177 amino acid positions in the alignment, presenting a large number of potentially adaptive substitutions. Only 5 of these 56 variable positions have been studied in the literature (Fig. 4; Table S1).

**Figure 5:**
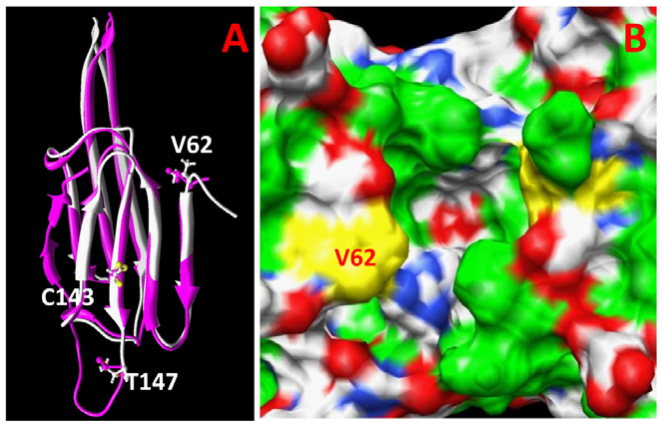
Structure of αA-crystallin domain, location of three modified sites and the effect of modification in position 62 are shown. (A) A superposition of monomeric molecules of a recent zebrafish crystal structure (PDB id: 3N3E) and the protein obtained using homology modeling with a bovine αA-crystallin structure (PDB id:3L1E) in this work are represented by magenta and white, respectively. Corresponding amino acid residues in zebrafish positions 62, 143 and 147 are shown by ball-and-sticks. (B) The accessible surface of the zebrafish homo-dimer (PDB id: 3N3E) with the non-polar surface shown in green. Non-polar surface of V62 is shown in yellow. This area will become polar when valine is replaced with a threonine residue.

To narrow down the possible number of functionally relevant changes, homology modeling of the zebrafish αA-crystallin sequence using the crystal structures of several non-vertebrate sHSPs was used to identify changes in residue hydrophobicity that might alter protein folding. This approach identified three putatively functional substitutions at zebrafish residues 62, 143, 147. After these positions were identified for the generation of site-directed mutants, as described below, several new mammalian α-crystallin structures were published (human: PDB id: 2WJ7, 3L1G), (rat: PDB id: 2WJ5) and (cow: PDB id:3L1E). The mammalian structures are very similar to a recent crystal structure for a partial zebrafish αA-crystallin (PDB id: 3N3E) (Fig. 5A). Stabilization energies calculated for each of the three putatively functional cold-adapted substitutions using this recent zebrafish αA-crystallin structure as a model were 0.44 kcal/mol (T147V), 0.54 kcal/mol (C143S) and 1.07 kcal/mol (V62T). These values are low and suggest that the three variants would not cause large changes in protein stability. However, homology modeling indicated that the wildtype zebrafish valine at position 62 is part of a large nonpolar pocket at the surface of the αA-crystallin domain (Fig. 5B). In large assemblies these pockets will likely be filled by hydrophobic sequence motifs from partner chains [36]. Substitution to a polar threonine in cold-adapted species at this position was predicted to decrease the binding of these hydrophobic chains and destabilize the oligomer. The cysteine residue at position 143 is also located on the α-crystallin domain surface. While replacement with a small polar serine residue in cold-adapted species could alter β-conformation of the forming β-strand residues 66–68, we predicted that the C143S variant would have less effect on overall protein stability. The substitution T147V in cold-adapted species disrupts three hydrogen bonds stabilizing the αA-crystallin dimer, but these are replaced by three new hydrogen bonds. Therefore, bonding alteration in the T147V variant was predicted to produce minor or no changes in structure. Overall, computer modeling predicted the greatest potential functional change in substitution V62T and the least in T147V.

### Altering hydrophobicity of specific zebrafish αA-crystallin residues changed chaperone-like activity and thermal stability

**Figure 6:**
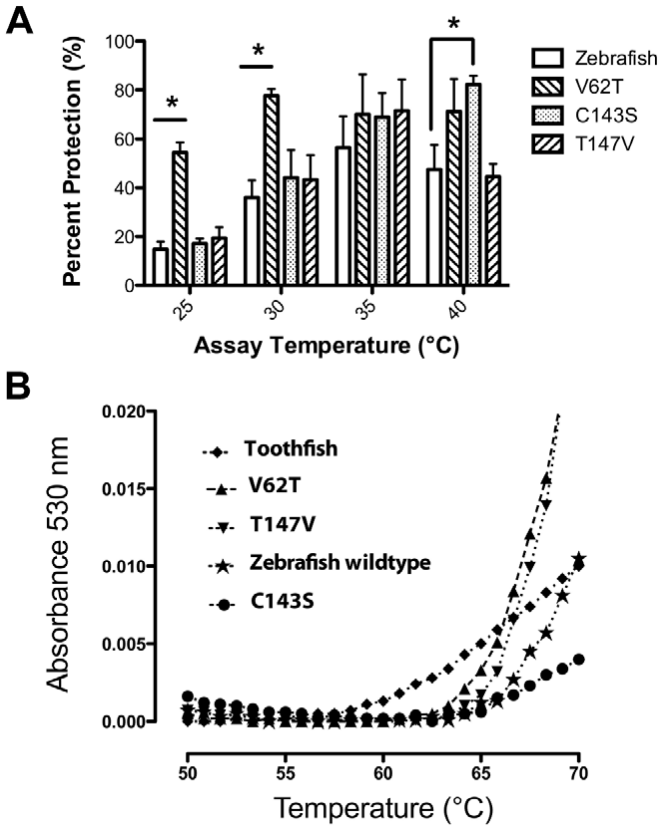
Effect of single amino acid substitutions on zebrafish αA-crystallin function and stability. (A) Two of the three single-point substitutions analyzed increased chaperone-like activity compared to wildtype zebrafish αA-crystallin when protecting against the chemically induced aggregation of lactalbumin. The V62T variant exhibited increased chaperone-like activity at 25° and 30°C while the C143S variant showed increased chaperone like-activity at 40°C. Asterisks indicate statistically significant differences (Two-way ANOVA with Bonferroni post-tests , p<0.05). The T147V variant showed little difference from the wildtype. Error bars indicate standard error of the mean (n = 3). (B) The V62T and T147V variants showed decreased thermal stability compared to wildtype zebrafish αA-crystallin while the C143S variant showed increased stability.

We used PCR techniques to produce zebrafish αA-crystallin cDNAs coding for proteins with each of the three separate amino acid substitutions described above reflecting residues found in the cold-adapted Antarctic toothfish (*Dissostichus*). Our hypothesis was that these substitutions would both decrease the upper level of thermal stability while increasing chaperone-like activity compared to wildtype zebrafish αA-crystallin. The V62T substitution fit this hypothesis, significantly enhancing chaperone-like activity at 25° and 30°C (Fig. 6A; p<0.05) and reducing the upper limit of thermal stability compared to the wildtype (Fig. 6B). The C143S substitution increased chaperone-like activity, but only at temperatures greater than 40°C (Fig. 6A; p<0.05) and increased thermal stability (Fig. 6B). The T147V substitution produced no significant change in chaperone-like activity at any temperature tested, but did exhibit a loss of thermal stability near 65°C (Fig. 6). Overall, one of the three modified αA-crystallins fit our hypothesis by exhibiting increased chaperone-like activity correlated with decreased upper limit of thermal stability, suggesting that substitutions at residue 62 are involved in the thermal adaptation of αA-crystallin. The other two substitutions (C143S and T147V) had effects on chaperone-like activity and thermal stability, but not in a correlated way, and less dramatically than the V62T substitution. Our functional results are consistent with predicted structure/function effects based on computer homology modeling from recent mammalian and zebrafish crystal structures [36], [37], [38].

**Figure 7:**
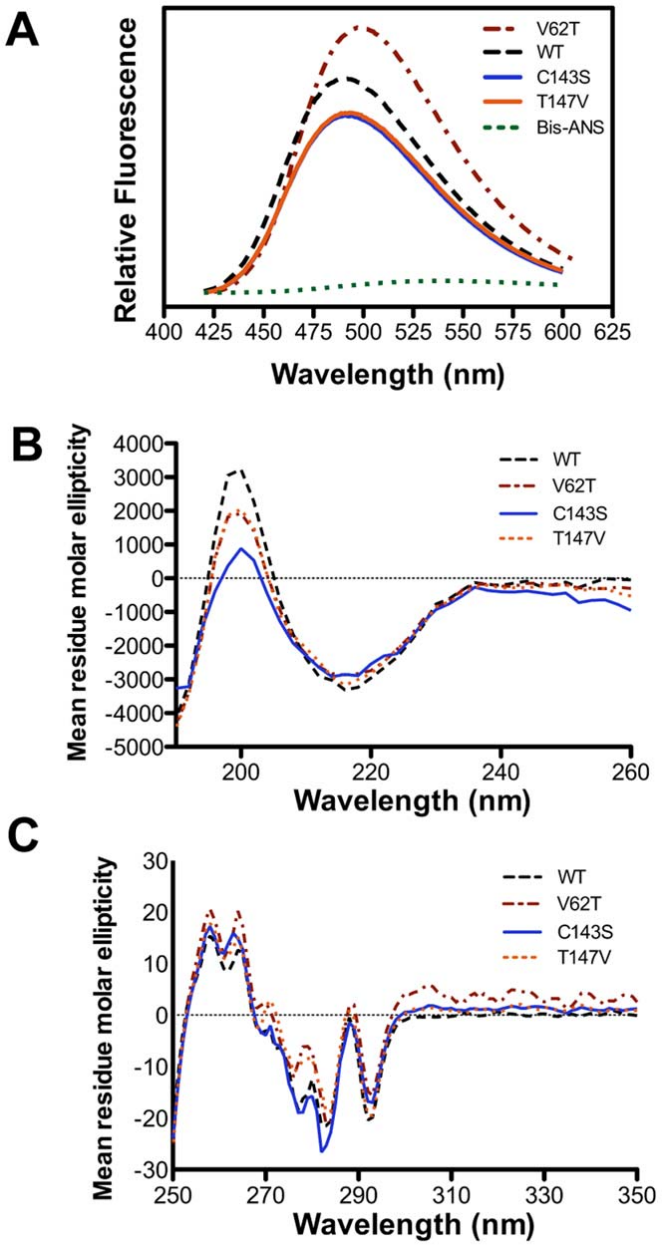
Surface hydrophobicity and circular dichroism spectroscopy of zebrafish αA-crystallin and three variants. Indicated in all panels are zebrafish αA-crystallin, V62T, C143S, and T147V variants. (A) Bis-ANS fluorescence spectra at room temperature indicates relative amount of surface hydrophobicity. Excitation wavelength was 390 nm and the protein concentration was 0.1 mg/mL. (B) Far UV CD spectroscopy indicating secondary structure shows a similar abundance of β-sheets in each protein. Scans were performed at 25°C. (C) Near UV CD spectroscopy, with deflections indicating the positions of aromatic amino acids in each tertiary structure. Scans were performed at room temperature.

Binding of the probe bis-ANS to wildtype and variant αA-crystallins was used to assess any changes in the quantity of exposed surface hydrophobicity. Bis-ANS binding increased in the V62T variant relative to the wildtype (Fig. 7A). This result is consistent with the increase in chaperone-like activity of the V62T variant, and suggests that reduced thermal stability caused by this single amino acid substitution may result in increased flexibility and exposure of hydrophobic surfaces. The C143S and T147V variants both showed decreased surface hydrophobicity from the wildtype (Fig. 7A). Difference between the wildtype and variants (C143S and T147V) was not correlated with chaperone-like activity, as these proteins showed no differences at 25°C, the temperature closest to the bis-ANS assays (Fig. 6A). Analysis by circular dichroism spectroscopy showed little difference between αA-crystallin and the three variants, indicating that secondary and tertiary structures are not altered by these modifications (Fig. 7B,C).

## Discussion

We previously showed that α-crystallin's protein binding chaperone-like activity and thermal stability have evolved to match physiological temperature [31], [34]. In this study we hypothesized that a comparison of fish species living in different environmental temperatures would permit identification of specific amino acid changes responsible for modifications in α-crystallin function. Data presented here support our hypothesis, and show for the first time that alterations in hydrophobicity are involved in the evolutionary adaptation of a sHsp to different physiological temperatures. Our results also show that comparisons between related species adapted to disparate thermal environments can be used to identify functionally relevant structural changes at the molecular level in sHsps, and potentially other globular proteins.

Determining the impact of hydrophobicity on α-crystallin function is complicated by its effects on both global stabilization and the hydrophobic interactions that attract α-crystallin subunits to denaturing target proteins [24]. For example, increased hydrophobicity can reduce chaperone activity by stabilizing α-crystallin oligomers and reducing subunit exchange, but alternatively promote chaperone binding if those hydrophobic residues are exposed on the chaperoning subunit. These opposite hydrophobic effects are further complicated by the impact of temperature. Our homology modeling suggests that much of the αA-crystallin surface hydrophobicity might exist in shallow depressions that would be shielded by motion at the protein surface (Fig. 5). Increased surface motion at higher temperatures could decrease the interactions between these hydrophobic regions and target proteins. Therefore, while rising temperatures will increase chaperone-like activity by promoting subunit exchange, it might also limit target protein binding by obscuring surface hydrophobicity. A comparison of multiple species from different environmental temperatures provides a useful model for dissecting the interacting effects of temperature and hydrophobicity on the evolution of αA-crystallin's chaperoning mechanism.

The data reported here show that thermal stability and chaperone-like activity are correlated with environmental temperatures, and have both likely adapted to differing physiological temperatures. While results from our V62T zebrafish variant indicate that a decrease in stabilization and increase in chaperone-like activity can be linked, several lines of evidence suggest that stability and chaperone-like activity can change independently as well. First, the *Pimephales* and *Oncorhynchus* αA-crystallins have different thermal stabilities (Fig. 3A) but very similar chaperone-like activities (Fig. 2). Second, the zebrafish T147V variant produced no significant change in chaperone-like activity, but did lead to a reduction in thermal stability. And third, the C143S variant led to increased chaperone-like activity at high temperatures with a modest increase in thermal stability. Overall these data fit a model in which global stability and chaperone-like activity can evolve independently, suggesting that different contributors to chaperone-like activity could be targets for selection. Previous studies on catalytic enzymes have also reported changes in function without alterations to global stability [39], [40]. The retention of normal secondary and tertiary structures in our variants (Fig. 7) highlights the benefit of examining naturally evolved proteins, where altered function can be identified without sacrificing the required structure for normal chaperone activity, as seen in some disease causing mutant sHsps [41].

It is worth noting that while our analysis suggests that the three substitutions tested in this study, especially the V62T variant, have played a role in the cold-adaptation of αA-crystallin, these changes are occurring in a global context with multiple other substitutions. It is possible that the specific single amino acid substitutions examined do not contribute to cold-adaptation when considered along with other changes to the protein sequence. Nevertheless, at a minimum our comparative approach was successful in identifying structural changes that modify sHsp function in a predictable way. Our findings are also consistent with a history of studies that have identified strong adaptive effects of single or relatively few amino acid changes in proteins as diverse as hemoglobin [42], [43], sodium channels [44], lactate dehydrogenase [45], [46] and malate dehdrogenase [47].

The diversity of phenotypic effects produced by the three hydrophobic substitutions in this study highlight the complex ways that hydrophobicity can alter sHsp function. Furthermore, the reduction in hydrophobic residues in warmer adapted αA-crystallins (Fig. 3C) shows that these proteins do not fit the typical adaptive strategy of using increased hydrophobicity to stabilize their internal core [48]. While the warmer-bodied αA-crystallins may draw more stabilization from each hydrophobic residue, it is also possible that the larger numbers of hydrophobic residues in cold-adapted αA-crystallins increase the protein binding necessary for chaperone-like activity at lower temperatures. By focusing on changes in hydrophobicity that might affect oligomer stability our approach has potentially overlooked increases in hydrophobicity that specifically enhance target protein binding. However, future studies can use the amino acid sequence alignment presented here to test the hypothesis that increased hydrophobicity at individual residues is important for cold adaptation of sHsps. Identification of these regions would allow the design of sHsps with stronger chaperone-like activity while keeping global stability constant. Strong TreeSAAP scores in the N-terminus region (Fig. 3B, 4), which is thought to affect sHsp oligomerization [49], suggest that changes in the hydrophobicity of this region may also play a role in thermal adaptation.

Despite a voluminous literature on α-crystallin structure/function relationships, this is the first study to use a comparative approach to identify functionally important changes in amino acid sequence and indicate the importance of hydrophobicity in the thermal adaptation of sHsps. This comparative approach complements the functional study of sHsp mutations found in many protein aggregation pathologies. Analyzing changes in naturally evolved proteins allows the characterization of modifications within the context of a normally functioning wildtype protein. While increased chaperone-like activity in some sHsp mutants can promote protein aggregation [50], thermally adapted wildtype proteins may suggest ways that increased activity could be used to improve protection against protein aggregation *in vivo*. Future work using the protein sequences from this study, as well as those from additional species, have the potential to provide more insights into the basic mechanisms behind sHsp function and evolution, and possible therapeutic uses of altered sHsps.

## Materials and Methods

### Cloning of αA-crystallins, site-directed mutagenesis and purification of recombinant protein

αA-crystallins from *Cyprinodon*, *Oncorhynchus* and *Pimephales* were cloned by first collecting total RNA from homogenized lens tissues using an RNEasy kit (Qiagen, Valencia, CA). RT-PCR amplification with primers (sense - 5′ TACCCCACCCGACTCTTTGA 3′; antisense 5′ ACATTGGAAGGCAGGCGGTA 3′) specific to a conserved region of the previously cloned zebrafish αA-crystallin [32] was used to produce an internal segment of each gene. Cloning was completed by 3′ and 5′ RACE using the RACE system for Rapid Amplification of cDNA ends (Invitrogen, Carlsbad, CA) with primers specific to each species. Clones for *Danio*, *Dissostichus* and *Notothenia* were produced previously [32], [51]. Full length coding sequences for each species' αA-crystallin gene were PCR amplified to incorporate a 3′ NDE1 site and 5′ BAMH1 site for ligation into the pET20b(+) expression vector (Novagen, Madison, WI), which was then used to transform BL21(DE3) cells (Novagen). Protein induction, cell lysis and purification were performed as described by Horwitz *et al*. [52] with the following modifications: 500 ml of liquid TB medium were inoculated with 10 ml of an overnight liquid culture of BL21(DE3) cells containing each expression construct. Liquid cultures were incubated at 37°C while shaking at 250 rpm until culture densities reached an absorbance of 0.4 at 500 nm, and then protein expression was induced with IPTG (final concentration 0.5 mM). Cell lysates were loaded onto a Mono-Q Hi Trap column (GE Healthcare, Piscataway, NJ) and eluted at 3 ml min^−1^ with 20 mM Tris, pH 8.5 with stepwise concentrations of 0.1 M, 0.2 M and 0.3 M NaCl. Fractions from the 0.3 M NaCl buffer containing αA-crystallin were concentrated to 1 ml in Amicon centrifugal filters (30,000 MW cutoff; Millipore, Billerica, MA) and further purified on a 90 cm×2.5 cm size exclusion column containing Sephacryl S-200 High Resolution bedding material (GE Healthcare) at a flow rate of 0.4 ml/min at 8°C. Fractions containing purified αA-crystallin were concentrated to approximately 5 mg/ml using Amicon centrifugal filters (30,000 MW cutoff; Millipore) and concentration was determined by spectrophotometry (1 OD_280_ = 1 mg ml^−1^). The purity of each purified protein was assessed by SDS-PAGE. Animal use in this study was approved by Ashland University's Institutional Animal Care and Use Committee (permit # 6).

### Determination of chaperone-like activity and thermal stability

Chaperone-like activity of each αA-crystallin was assayed be measuring their ability to prevent the chemically induced aggregation of insulin and/or lactalbumin. These target proteins were used because they aggregate across a broad range of temperatures (20 to 40°C) when chemically denatured with DTT. Other commonly used target proteins, such as lysozyme, do not aggregate well at temperatures below 30°C. Neither target protein used aggregated well below 20°C or above 40°C, so this was the range used in the assays. Three replicates were performed with all αA-crystallins at each temperature for both a-lactalbumin (Sigma L6010; 0.6 mg ml^−1^) and insulin (Sigma I5500; 0.3 mg ml^−1^) as the target protein. Insulin assays were performed with a mass ratio of 3∶1 target to crystallin in a buffer containing 50 mM sodium phosphate, 0.1 M NaCl, pH 7.0. The insulin was dissolved in 0.1 N NaOH, and 0.5 M NaPO_4_ (pH 6.8) was added to raise pH to 7.7. Lactalbumin assays used either a 10∶1 or 20∶1 ratio of target to crystallin in a buffer containing 50 mM sodium phosphate, 0.1 M NaCl, pH 6.75. Lactalbumin was dissolved directly in this buffer prior to use. Aggregation was induced with 20 mM DTT and monitored by measuring light scattering at 360 nm on a Shimadzu UV 1601 with a CPS 240 A temperature controller. All reactions were in a total of 500 µl using a 5 mm path length cuvette. Percent protection was calculated from the assay data by measuring the proportion of target protein aggregation prevented by each crystallin. Two way ANOVA was used to calculate statistical differences between αA-crystallins (GraphPad Prism; http://www.graphpad.com).

The relative thermal stability of each native and modified αA-crystallin was tested by measuring the point at which thermally induced aggregation occurred under increasing temperatures. For each assay, 0.2 mg ml^−1^ of each purified crystallin in 50 mM sodium phosphate, 0.1 M NaCl, pH 7.0 was placed in a series of 0.5 ml cuvettes in a Shimadzu UV 1601 with a CPS 240 A temperature controlled cuvette block. The cuvettes and crystallin solutions were heated to 50°C and then temperature was increased 2.5°C every 15 minutes. Light scattering was measured at either 360 or 530 nm throughout the experiment to determine when protein aggregation began.

### Analysis of αA-crystallin alignment and homology modeling of αA-crystallin structure

Multiple sequence alignments were produced with ClustalW [53] using Biology Workbench (http://workbench.sdsc.edu/). Structure of the bovine αA-crystallin (PDB ID: 3L1E) was used as the structural template to model protein structures of six fish αA-crystallins. Structure visualization was performed using the module incorporated in the UCSF Chimera, build 1.4.1 [54], and primary sequences were aligned using the method of Needleman and Wunsch [55] integrated in the program Look, version 3.5.2 [56], [57] for tertiary structure prediction. The location of the major functional components of α-crystallin was predicted by SMART [58], [59]. The α-crystallin domains were built by the automatic segment matching method in the program Look [60], followed by 500 cycles of energy minimization. The conformation of the 3 missense variants of zebrafish α-crystallin (PDB ID: 3N3E), V62T, C143S and T147V, was generated by the same program implicating a self-consistent ensemble optimization (500 cycles) [56].

Structures of wild type proteins and the zebrafish missense variants were refined using the minimization procedure performed with the Impact module of the Maestro program package (version 8.0.308, Schrodinger, Inc., New York, NY, USA). Hydrogen atoms were added to the structures. Protein structures were regularized by an energy minimization procedure using the OPLS_2005 potentials, the 12 Å non-bonded cut-offs, the distance-dependent dielectric constant and 100 steepest descent steps of minimization followed by 200 steps of conjugated gradient in the presence of explicit 7453 SPC water molecules in the 50 Å×70 Å×70 Å on the final step. All bonds were constrained by the linear constraint solver algorithm. Temperature was kept constant to 298.15 K. Finally, the quality of modeled structures tested with the program Procheck [61].

The evaluation of protein stabilization free energy for the six fish α-crystallin domains was accessed with the FoldX force field, based on an empirical energy function derived from experimental work on proteins [62], [63]. The FoldX free energy is a combination of solvation energy contributions from hydrophobic and polar groups of protein; a van der Waals energy term taking into account experimental transfer energies from water to vapor; the energy of hydrogen bonds with regards to simple geometric considerations; electrostatic coulomb free energy terms; and the crude entropy of protein chains to obtain a measure of free energy and free energy of the steric overlaps between atoms in protein structure.

### Analysis of molecular adaptation

For phylogenetic reconstruction, αA-crystallins from six ectothermic fish species and endothermic human (see Figure 5 for GenBank accession nos.) were first aligned using ClustalW (Hall, TA 1999) by *in silico* translated amino acid sequences. Corresponding nucleotide sequences were then aligned by codon constraint to each other using the amino acid alignment via CodonAlign 2.0 [64], and tree construction was done by MrBayes 3.1.2 using the evolutionary model GTR+I [65], as selected by MrModelTest v2.3 [66]. Four runs using four chains and 1,000,000 generations were performed. All other parameters in MrBayes 3.1.2 were left at default. Stationarity was assessed using the “sump burnin = 250" command and examining the plot of the generation versus the log likelihood values. A 50% majority rule consensus tree was generated using the command “sumt burnin = 250".

Molecular adaptation analysis was performed using TreeSAAP 3.2 [67] that implements PAML 3.14 [68], [69] and selected biochemical properties (for description of TreeSAAP and its usage, see McClellan *et al*. [70] and http://tinyurl.com/treesaap). Because we were aware that hydrophobicity and chaperone properties are likely linked [27], [71], [72], [73], we choose to analyze the αA-crystallins in terms of hydrophobic properties. Essentially, TreeSAAP uses a nucleotide alignment and a phylogram (in our case the Bayesian 50% MJ rule consensus tree described above) given to it to reconstruct ancestral sequences using the PAML code [69]. TreeSAAP then evaluates changes in molecular adaptation, codon by codon, from the ancestral sequences to the extant sequences in the context of a PAML generated phylogenetic topology and estimates the effect of codon replacements events based on up to 516 different physiochemical properties of replaced amino acids using a sliding window approach [74]. TreeSAAP scores these changes on a magnitude scale (1 to 8) with eight being the most radical or destabilizing, and one being the least radical, or stabilizing replacement events [70], [74]. Goodness-of-fit and other statistical analysis such as each magnitude receiving a Z-Score that indicates the likelihood of the change being due to neutral evolution or to positive selection; Z-Scores of 1.64 are at the P = 0.05, 2.32 at P = 0.01 and 3.09 at P = 0.001 significance levels (respectively).

### Measurement of surface hydrophobicity and protein structure

Fluorescence emission spectra were recorded with a Cary Eclipse fluorescence spectrophotometer (Varian, Agilent Technologies). Protein samples of 0.1 mg/mL in 10 mM phosphate buffer, pH 7.0, were used. Excitation was fixed at 295 nm and emission scanned from 310 to 400 nm. Binding of the hydrophobic probe bis-ANS (4,4′-dianilino-1,1′-binaphthyl-5,5′-disulfonic acid, dipotassium salt) (Molecular Probes, Invitrogen) was assessed by recording fluorescence spectra with excitation wavelength fixed at 390 nm and emission spectra recorded between 420 and 600 nm as follows: 10 µL of 20 mM methanolic solution of Bis-ANS was added to a 1 mL protein (0.1 mg/mL) solution in 10 mM phosphate buffer, pH 7.4, and incubated for 2 h.

To investigate secondary and tertiary structural changes of recombinant zebrafish αA-wild-type and our three variants their near- and far-UV CD spectra were recorded using a Jasco 500 A spectropolarimeter. Near-UV CD spectra were recorded with protein concentrations of 2.8, 2.0, 2.8, and 2.1 mg/mL, respectively, in 10 mM potassium phosphate buffer, pH 7.4 at room temperature. The reported CD spectra are the average of seven smoothed scans with an optical path of 1 cm. Far-UV CD spectra were recorded with protein concentrations of 0.09, 0.05, 0.04, 0.04 mg/mL, respectively, in 10 mM potassium phosphate buffer, pH 7.0 at 25°C. The reported spectra are the average of 12 smoothed scans with an optical path of 0.2 cm.

## Supporting Information

Table S1 **αA-crystallin modifications previously reported in the literature.** Modified αA-crystallins and their corresponding chaperone-like activity are shown. Each of these modifications were produced in mammalian αA-crystallins. The three variants produced in this study are novel in that their functional effects were predicted by comparing amino acid sequences from six different species.
(DOC)
