# Supplementary material for: Functional Validation of Hydrophobic Adaptation to Physiological Temperature in the Small Heat Shock Protein αA-crystallin
Source: PLoS One. 2012 Mar 29;7(3):e34438. doi: 10.1371/journal.pone.0034438 (PMC3315530; doi:10.1371/journal.pone.0034438)
Supplement: Table S1 — αA-crystallin modifications previously reported in the literature. Modified αA-crystallins and their corresponding chaperone-like activity are shown. Each of these modifications were produced in mammalian αA-crystallins. The three variants produced in this study are novel in that their functional effects were predicted by comparing amino acid sequences from six different species. (DOC) [file pone.0034438.s001.doc]

**Supplemental** **Table 1. αA-crystallin modifications previously reported in the literature.**

| **Modification** | **Chaperone Activity** | **Reference** |
| --- | --- | --- |
| R12C | Decrease | [1] |
| R21A | Increase | [2, 3] |
| R21A/R49A | Decrease |
| R21A/R103A | Increase |
| R21A/R49A/R103A | Increase |
| R49A,C | Both Decrease |
| R103A | Increase |
| R54C | Decrease | [4] |
| D69S | Decrease | [5] |
| F71G | Decrease | [6] |
| F71L | Decrease | [7] |
| G98R | Decrease | [8] |
| N101D | Decrease | [9] |
| N101D/N123D | Decrease |
| N123D | Decrease |
| R116C,H,G,D | All Decrease | [10-12] |
| R116K | No Change |
| R116C/S142C | Decrease |
| S142C | No Change |
| Y118D | Decrease | [4] |
| C131I | Decrease | [13] |
| C131I/C142I | Decrease |

Modified αA-crystallins and their corresponding chaperone-like activity are shown. Each of these modifications were produced in mammalian αA-crystallins. The three variants produced in this study are novel in that their functional effects were predicted by comparing amino acid sequences from six different species.

**References**

1. Zhang L-Y, Yam GH-F, Tam PO-S, Lai RY-K, Lam DS-C, et al. (2009) An alphaA-crystallin gene mutation, Arg12Cys, causing inherited cataract-microcornea exhibits an altered heat-shock response. Mol Vis 15: 1127-1138.

2. Biswas A, Miller A, Oya-Ito T, Santhoshkumar P, Bhat M, et al. (2006) Effect of site-directed mutagenesis of methylglyoxal-modifiable arginine residues on the structure and chaperone function of human alphaA-crystallin. Biochemistry 45: 4569-4577.

3. Mackay DS, Andley UP, Shiels A (2003) Cell death triggered by a novel mutation in the alphaA-crystallin gene underlies autosomal dominant cataract linked to chromosome 21q. Eur J Hum Genet 11: 784-793.

4. Xia C-h, Liu H, Chang B, Cheng C, Cheung D, et al. (2006) Arginine 54 and Tyrosine 118 residues of {alpha}A-crystallin are crucial for lens formation and transparency. Invest Ophthalmol Vis Sci 47: 3004-3010.

5. Smulders RH, Merck KB, Aendekerk J, Horwitz J, Takemoto L, et al. (1995) The mutationASP69 - Ser affects the chaperone-like activity of αA-crystallin. European Journal of Biochemistry 232: 834-838.

6. Santhoshkumar P, Sharma KK (2001) Phe71 is essential for chaperone-like function in alpha A-crystallin. J Biol Chem 276: 47094-47099.

7. Bhagyalaxmi SG, Srinivas P, Barton KA, Kumar KR, Vidyavathi M, et al. (2009) A novel mutation (F71L) in alphaA-crystallin with defective chaperone-like function associated with age-related cataract. Biochim Biophys Acta 1792: 974-981.

8. Singh D, Raman B, Ramakrishna T, Rao CM (2006) The cataract-causing mutation G98R in human alphaA-crystallin leads to folding defects and loss of chaperone activity. Molecular vision 12: 1372-1379.

9. Gupta R, Srivastava OP (2004) Deamidation affects structural and functional properties of human alphaA-crystallin and its oligomerization with alphaB-crystallin. J Biol Chem 279: 44258-44269.

10. Shroff NP, Cherian-Shaw M, Bera S, Abraham EC (2000) Mutation of R116C results in highly oligomerized alpha A-crystallin with modified structure and defective chaperone-like function. Biochemistry 39: 1420-1426.

11. Bera S, Abraham EC (2002) The alphaA-crystallin R116C mutant has a higher affinity for forming heteroaggregates with alphaB-crystallin. Biochemistry 41: 297-305.

12. Pang M, Su J-T, Feng S, Tang Z-W, Gu F, et al. (2010) Effects of congenital cataract mutation R116H on alphaA-crystallin structure, function and stability. Biochimica et biophysica acta 1804: 948-956.

13. Chen SJ, Sun TX, Akhtar NJ, Liang JJ (2001) Oxidation of human lens recombinant alphaA-crystallin and cysteine-deficient mutants. J Mol Biol 305: 969-976.
